# Simulation and Process Optimization of Online Cooling for S460 Thick Plates

**DOI:** 10.3390/ma18112599

**Published:** 2025-06-03

**Authors:** Guangyuan Wang, Zhen Wang, Feng Chai, Zhongwen Wu, Xiaobing Luo, Tao Pan

**Affiliations:** 1 Division of Structural Steels, Central Iron and Steel Research Institute, Beijing 100081, China; wgydslazy777@163.com (G.W.); chaifeng666@vip.sina.com (F.C.); luosir2007@sina.com (X.L.); 2 Technology Center, Hunan Valin Xiangtan Iron and Steel Co., Ltd., Xiangtan 411101, China; even320@163.com (Z.W.); m15973218185@163.com (Z.W.)

**Keywords:** microstructure, mechanical properties, finite element simulation, process optimization

## Abstract

Marine engineering thick plates are essential structural materials for large vessels and offshore platforms, and optimizing their manufacturing processes is critical for advancing marine equipment. This study examined the microstructural and property variations in 120 mm-thick S460 plates fabricated by thermo-mechanical controlled processing (TMCP). A finite element model was developed to simulate the cooling phase, enabling the prediction of the internal cooling path in the thick plate. An optimized cooling scheme was proposed, which was validated against the model and implemented. The following key results were obtained: (1) Under the initial cooling parameters (initial temperature: 715 °C, duration: 130 s), the 60 mm depth toughness was severely compromised, as evidenced by a low −40 °C impact energy of 59 J, significantly lower than values observed at the10 mm and 30 mm depth positions. Microstructural analysis revealed that the 60 mm depth region was dominated by ferritic bainite and pearlite, with a pearlite content of 8.7%. Numerical simulations further indicated a 60 mm depth cooling rate of 1.10 °C/s under these conditions. (2) Model predictions confirmed the original 60 mm depth cooling rate of 1.10 °C/s. The optimized process increased the initial cooling temperature to 725 °C and extended the cooling time to 160 s, achieving an enhanced 60 mm depth cooling rate of 1.36 °C/s. (3) The optimized process remarkably improved the 60 mm depth impact energy to 144 J, achieving near-complete elimination of pearlite, increased granular bainite content, refined M-A constituent size, and enhanced density of high-angle grain boundaries. This study demonstrates that enhancing internal temperature gradients and prolonging cooling durations can effectively inhibit microstructural degradation in 60 mm depth regions of thick plates, providing both theoretical foundations and practical methodologies for optimizing TMCP processes of extra-thick steel plates.

## 1. Introduction

In recent years, marine resource exploitation has expanded to more extreme latitudes and deeper ocean regions [1,2,3], increasing the demand for thick steel plates in offshore engineering. These plates form the structural backbone of large marine vessels and platforms and require a uniform microstructure with superior mechanical properties [4]. However, as the plate thickness increases, performance degradation at the center becomes a concern, potentially compromising the functionality of the plate [5]. Research has shown that insufficient cooling rates at the center of thick plates during cooling can result in microstructural degradation, which is a key factor in their reduced performance [6,7]. Matthias et al. [8] demonstrated that size effects due to cooling rate variations significantly influence martensitic transformation kinetics in low-alloy steels, which aligns with the challenges observed in thick plate manufacturing. Liu et al. [9] attributed this issue to the low density of large-angle grain boundaries in the center of thick plates, resulting in decreased impact toughness. Cui et al. [10] proposed that grain size and carbide distribution were the predominant factors influencing the strength and toughness of thick plates, while Zhang et al. [11] demonstrated that varying phase transformation types across different regions of thick plates resulted in mechanical property disparities. While these studies identified the causes of inferior mechanical properties at the center of thick plates, no optimization strategies have been proposed.

Thermo-mechanical controlled processing (TMCP) is a widely used method for producing thick plates [12,13], with its controlled cooling stage playing a crucial role in the solid-state phase transformation of supercooled austenite, which directly affects the performance of the final product [14,15]. However, monitoring the temperature during cooling in industrial production is inherently challenging, necessitating the use of the return-back temperature, which is measured on the surface of thick plates at a specific time after water cooling stops [16,17]. However, this method often fails to precisely control the microstructure throughout the thickness of the plates. Therefore, accurately predicting the cooling conditions across different sections of thick plates is essential. Most existing studies have determined the transfer coefficients using empirical formulas and simulation experiments, which were then used to construct finite element models. However, the predictive accuracy of these models remains limited [18,19,20,21,22,23], where dynamic cooling conditions (e.g., water flow variability and surface oxidation) and nonlinear thermophysical properties during phase transformations introduce significant uncertainties. Empirical formulas often oversimplify convective heat transfer by assuming steady-state conditions or neglecting localized cooling inhomogeneities, while thermophysical parameters (e.g., thermal conductivity and specific heat) are typically averaged rather than dynamically adjusted. Machine learning provides an alternative approach for constructing cooling models. Xie et al. [24] proposed a machine learning-based transient heat transfer model for moving quenching jets, which could inspire refined cooling control in thick plate production. Stuebner et al. [25] employed physics-informed machine learning to reconstruct temperature fields during water quenching, achieving high predictive accuracy. However, it requires substantial datasets. Therefore, developing a more precise and practical model for predicting the cooling temperature field of thick plates is critical for optimizing cooling processes during thick plate manufacturing.

This study examined the microstructural evolution across the cross-section of a 120 mm-thick S460 plate. By tracking the production process, a novel approach was proposed for constructing a heat transfer model for thick plates. This method leverages the reheating process to correct the convective heat transfer coefficients during the cooling stage. The model was then employed to predict the cross-sectional temperature field during the cooling process of TMCP thick plates. Subsequently, the reliability of the model was validated by comparing the predicted results with the measured data and cross-sectional microstructures, showing improved accuracy compared to conventional prediction methods. Based on this validated model and the material characteristics, the TMCP was optimized to obtain more uniform cross-sectional microstructures and enhanced properties at a depth of 60 mm.

## 2. Materials and Methods

### 2.1. Materials

**Figure 1:**
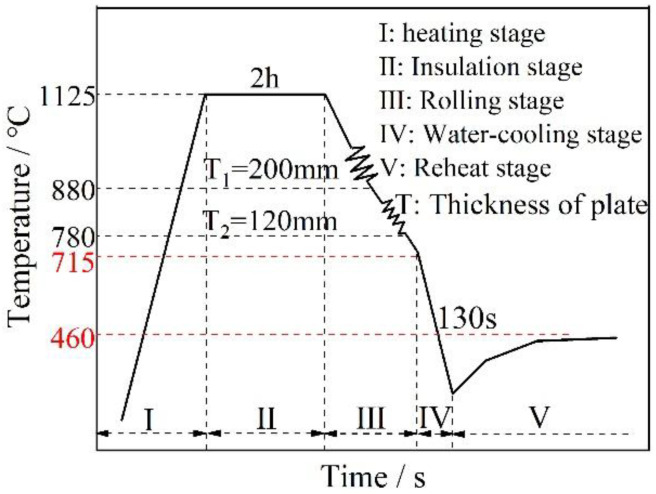
Production process of the thick plate.

The experimental material consisted of a 120 mm-thick S460 plate produced by a steel mill, with its chemical composition presented in Table 1. The production process for the thick plate is shown in Figure 1. First, a 450 mm-thick slab was heated to 1125 °C and maintained at this temperature for sufficient soaking. Subsequently, the slab was subjected to rough rolling to obtain an intermediate slab and reduce its thickness to 220 mm. Further rolling in the finishing mill decreased the thickness to 120 mm, with rolling beginning at 880 °C and ending at 780 °C. Before entering the accelerated cooling stage, the surface temperature of the steel plate was 715 °C. The plate was then rapidly cooled for 130 s using a MULPIC-controlled cooling system (for Siemens VAI, Linz, Austria). After cooling was stopped, the thick plate was reheated, and the reheating temperature was recorded at a fixed point of 460 °C. The initial cooling temperature was measured using a Raytek RAYCML3 infrared thermometer (for Raytek Corporation, Santa Cruz, CA, USA), and the outlet water temperature was measured using an Optris CTlaser LT (for Optris GmbH, Berlin, Germany), both integrated into the MULPIC system. The temperature recovery phase temperatures were acquired on-site using an Optris OPTP20LT handheld thermometer (for Optris GmbH, Berlin, Germany).

**Figure 2:**
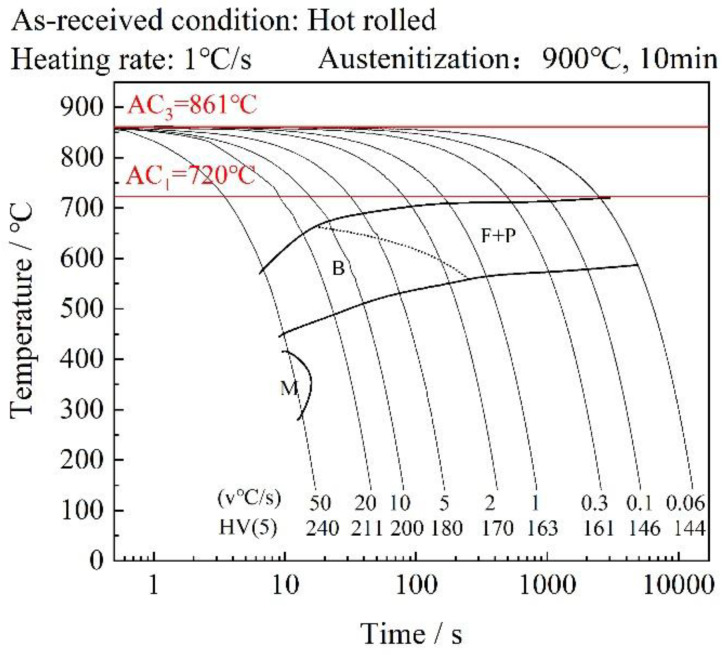
Continuous cooling transformation diagram of S460.

The phase transformation behavior of the investigated steel was characterized using a Formastor-Digital phase transformation analyzer (for Fuji Electronic Industrial Co., Ltd., Tokyo, Japan). The specimens were austenitized at 900 °C for 10 min, followed by continuous cooling at 0.1–20 °C/s. Through analysis of thermal expansion curves and corresponding derivative plots, phase transition temperatures were precisely identified. The established continuous cooling transformation (CCT) diagram (Figure 2) integrates dilatometric data with metallographic characterization and hardness measurements, comprehensively revealing the cooling rate-dependent phase evolution. Martensite formation requires a critical cooling rate exceeding 20 °C/s for this type of steel. When the cooling rate was between 1.7 °C/s and 8.5 °C/s, the resulting mixed microstructure consisted of bainite and ferrite. This composite microstructure of hard and soft phases can maintain high strength while providing favorable overall mechanical properties.

### 2.2. Microstructure

After mechanical polishing and etching with a 4% nitric acid alcohol solution, the microstructures were observed and photographed using a Leica MEF4M optical microscope (OM) (for Leica Microsystems, Wetzlar, Germany) and a Quanta-650 field emission scanning electron microscope (FE-SEM) (for Thermo Fisher Scientific, Eindhoven, The Netherlands).

Electron backscatter diffraction (EBSD) analysis was performed using a JSM-7900F scanning electron microscope (SEM) (for JEOL Ltd., Tokyo, Japan) equipped with a Symmetry EBSD detector to capture the EBSD pattern information of the samples. The scanning step size for the EBSD was set to 0.2 μm. Sample preparation for EBSD analysis involved machining, grinding, and mechanical polishing, followed by electrochemical polishing in a 6% perchloric acid solution at a constant voltage of 18 V. The effective grain size (EGS) was determined using the line intercept tool in Channel-5 software (version 5.0), while the other EBSD data were analyzed using AZtec-crystal software (version 2.1).

### 2.3. Mechanical Properties

**Figure 3:**
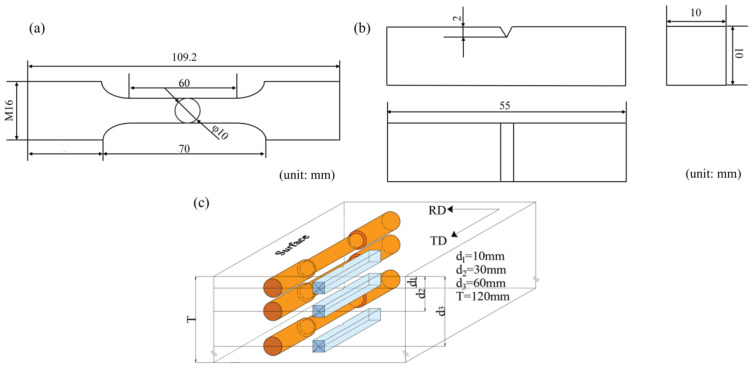
Schematic diagrams of mechanical property specimens: (**a**) tensile specimen, (**b**) impact specimen, and (**c**) sampling locations.

Low-temperature impact and room-temperature tensile tests were conducted on steel samples extracted from different positions on the plate. Due to the symmetry of the plate, three representative positions from the surface to the center were selected for testing. Tensile tests were carried out at 20 °C using specimens 10 mm in diameter, with the tensile axis perpendicular to the rolling direction. Impact tests were conducted at −40 °C using standard V-notched specimens. A schematic diagram illustrating the specimen dimensions and sampling locations is presented in Figure 3. To ensure reliable results and minimize experimental variability, triplicate samples were employed at each location.

### 2.4. Cooling Model

The model was developed using the thermal conduction module in the commercial finite element analysis software ABAQUS (version 6.14). The continuous temperature field was discretized by applying the heat conduction equation in conjunction with Newton’s law of cooling. The resulting differential equations were converted into equivalent integral equations using the method of variations. These integral equations were then discretized to derive an approximate temperature solution by solving the corresponding system of algebraic equations [26,27].

The precise determination of the convective heat transfer coefficient is crucial for model precision. In industrial production, this coefficient is typically estimated using empirical formulas that express it as a function of the water volume and temperature. However, these formulas often produce inconsistent predictive accuracies. To address this, the present study utilized temperature data from the air-cooling stage to refine the convective heat transfer coefficient for the preceding water-cooling stage, thereby predicting the cooling curves for both the water-cooling and air-cooling stages across the entire thickness of a plate. The detailed methodology for this adjustment is provided in Section 3.3.

## 3. Results

### 3.1. Mechanical Properties and Microstructures

**Figure 4:**
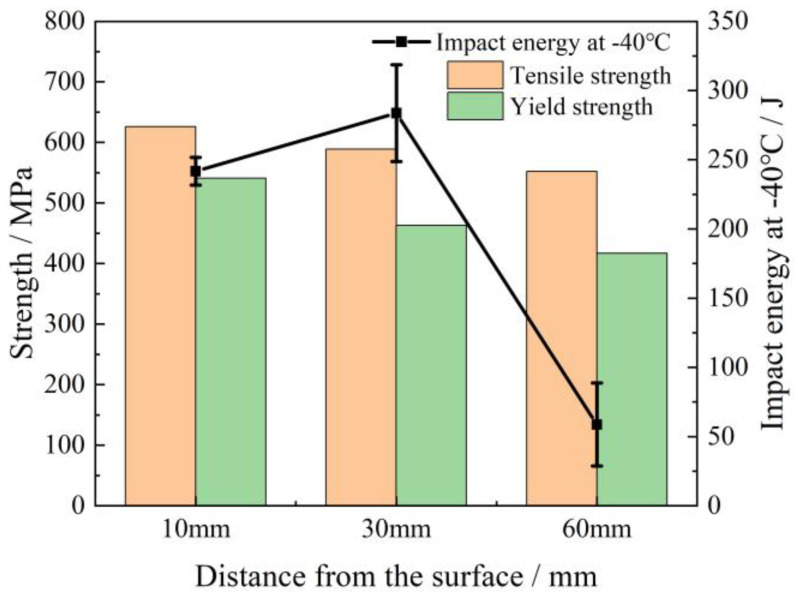
Mechanical properties at various locations of S460 thick plate.

Figure 4 presents the mechanical properties of the steel plate, indicating that the strength decreased from a depth of 10 mm to 60 mm. Specifically, the tensile strength decreased from 626 to 578 MPa, and the yield strength decreased from 532 to 428 MPa. The impact energy at the 30 mm depth reached the highest value of 283 kJ at −40 °C, followed by 241 kJ at the 10 mm depth. In contrast, the 60 mm depth exhibited a significant reduction in impact energy, with a value of only 59 kJ.

**Figure 5:**
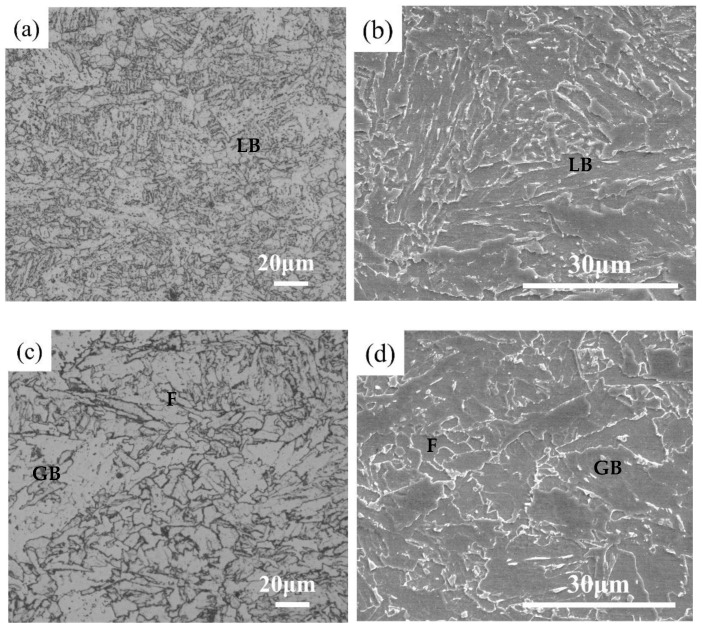

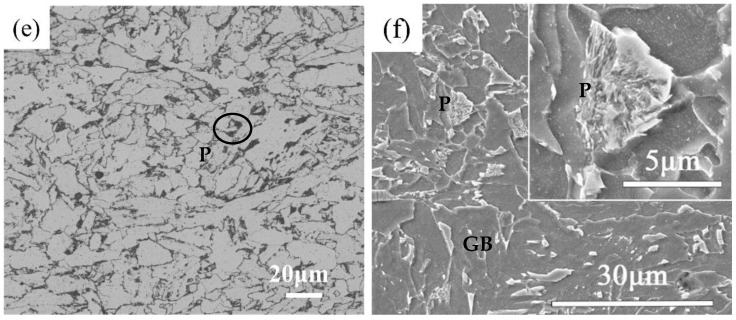
Microstructures at different locations in the S460 thick plate: (**a**,**b**): 10 mm depth (OM/SEM), (**c**,**d**): 30 mm depth (OM/SEM), (**e**,**f**): 60 mm depth (OM/SEM).

Figure 5 shows the microstructures at different cross-sectional positions. At a depth of 10 mm, the microstructure was mainly lath bainite (LB), while at a depth of 30 mm, it consisted of a mixture of granular bainite (GB), ferrite (F), and a small amount of lath bainite. At a depth of 60 mm, the microstructure primarily comprised a combination of ferrite and granular bainite, with a small amount of pearlite (P) present at the ferrite grain boundaries. The carbides in the pearlite exhibited an irregular fibrous morphology and were distributed near the ferrite grain boundaries.

### 3.2. Cooling Model of Thick Plate

#### 3.2.1. Thermal Physical Property Parameter Settings

**Figure 6:**
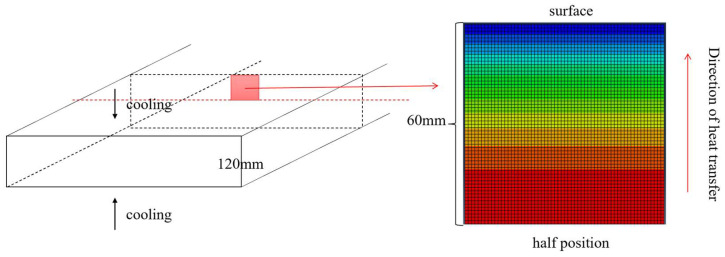
Schematic diagram of the thick plate heat transfer model.

The finite element model was developed through the following steps: geometric modeling, material property definition, boundary condition assignment, mesh generation, and solver configuration. Given the symmetry of the upper and lower surfaces, combined with the large width-to-thickness ratio of the plate, the heat transfer process was simplified as one-dimensional conduction from the core (60 mm depth) to the surface (0 mm depth). Consequently, the geometric model (Figure 6) was reduced to a one-dimensional representation. The surface (0 mm depth) was defined as the convective heat transfer boundary, and the remaining three faces were assigned adiabatic conditions. The heat generated within the plate propagated radially from the core (60 mm depth) to the surface (0 mm depth), where it dissipated into the ambient environment.

The finite element model was established through the following steps: model creation, material property definition, boundary condition setup, mesh generation, and analysis setup. Due to the symmetry of the upper and lower surfaces of the thick plate and the large width of the plate, the heat transfer process can be approximated as a one-dimensional heat transfer process from the center to the surface. Therefore, the geometric model of the thick plate heat transfer process can be simplified, as shown in Figure 6. The surface was designated as the heat-exchange interface, and the remaining three faces were treated as insulated boundaries. Heat within the thick plate was transferred from the center to the surface, dissipating into the external environment through the upper surface.

To simulate the heat transfer process, it was necessary to define the thermophysical properties of the material, including the density (ρ), specific heat (c), and coefficient of thermal conductivity (λ). Based on the relevant literature, the density of the steel was set to 7850 kg/m^3^, with the specific heat and thermal conductivity parameters provided in Table 2 [27]. The decrease in thermal conductivity at 500–600 °C (Table 2) aligns with the latent heat release during the austenite-to-bainite transformation, which temporarily reduces the heat dissipation efficiency. Furthermore, the abrupt increase in λ at 700 °C (Table 2) corresponds to the onset of ferrite formation, which enhances the thermal conductivity due to the reduced lattice distortion.

#### 3.2.2. Boundary Condition Settings

To develop an accurate heat transfer model, it is necessary to obtain the convective heat transfer coefficient during the cooling phase. In the cooling stage of the TMCP process, thick plates are often not completely cooled to completion but undergo rapid cooling for a set duration before the process is halted. The residual internal heat then causes the surface to reheat, inducing a self-tempering effect. Therefore, by tracking and recording the reheating process along with the initial conditions, the convective heat transfer coefficients for the rapid cooling and reheating phases can be calculated.

Based on on-site process tracking, the surface temperature of the thick plate was 715 °C when it entered the cooling device; however, when it was removed from the cooling device, the surface temperature was 280 °C, with a cooling time of 130 s. Using the removal time as the reference point (0 s), the surface reheating data were recorded and are listed in Table 3.

According to the reheating data, we observed that within 360 to 600 s after cooling stopped, the surface temperature of the thick plate remained around 470 °C. This temperature was the result of the combined effects of the rapid cooling phase and reheating phase. The reheating phase, which involved air cooling, led to minimal heat loss compared to the rapid cooling phase, and the calculation error in this process had a minimal impact on the accuracy of the model. Therefore, to simplify the calculations, the convective heat transfer coefficient for the air-cooling phase (h_A_) was calculated using the following empirical formula [28]:

(1) $$h=2.25{\left(T_{W}-T_{C}\right)}^{0.25}+4.6\times 10^{-8}({T_{W}}^{2}-{T_{C}}^{2})(T_{W}+T_{C})$$

where *T_W_* is the surface temperature of the plate, while *T_C_* is the ambient temperature. The convective heat transfer coefficient associated with air cooling was determined as 15 W/(m^2^·°C).

In terms of the convective heat transfer coefficient during the rapid cooling phase, we initially relied on a conventional calculation approach, which estimated the coefficient using the following empirical formula [28]:

(2) $$h=225.9\omega^{0.646}\times 1.163\;(T_{0}<200\;{}^{\circ}C)$$

(3) $$h=494.3\omega^{0.595}\times 10^{-0.00179T_{0}}\times 1.163\;(200\leq T_{0}<500\;{}^{\circ}C)$$

(4) $$h=107.2\omega^{0.663}\times 10^{-0.00147T_{0}}\times 1.163\;(T_{0}\geq 500\;{}^{\circ}C)$$

where *h* is the heat transfer coefficient, and ω is the channel frequency.

**Figure 7:**
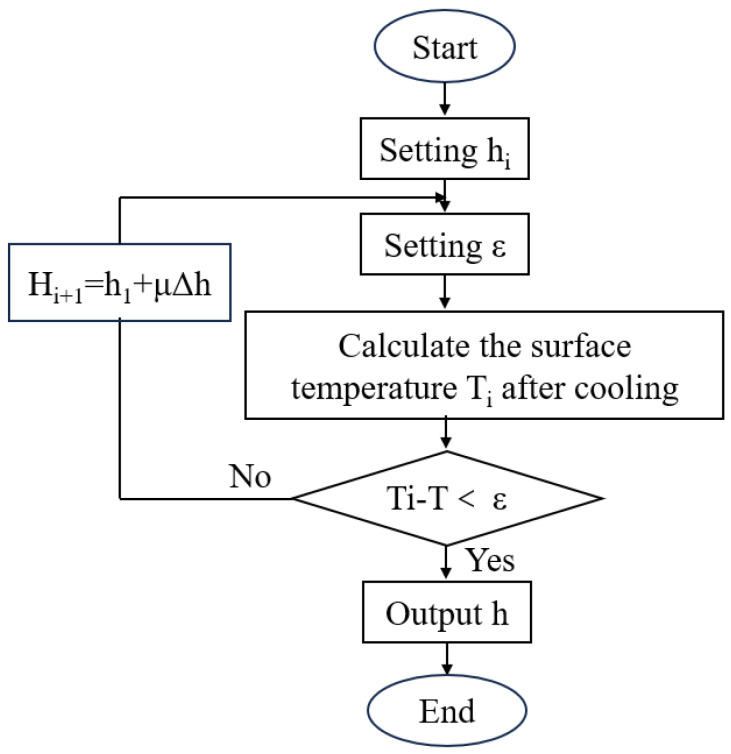
Heat transfer coefficient correction process.

To obtain a more accurate heat transfer coefficient, in this study, we corrected the results calculated from the empirical formula. The calculation flowchart is shown in Figure 7. The cooling process was initially simulated using the existing heat transfer coefficient to calculate the surface temperature curve, and the temperature difference between the simulated results and measured temperature points was determined. When this difference exceeded the preset error tolerance coefficient (*ε*), the convective heat transfer coefficient was adjusted and recalculated. This iterative process continued until the discrepancy between the predicted and measured data was less than *ε*. The convective heat transfer coefficients before and after correction are shown in Table 2. The original heat transfer coefficient (h) derived from empirical formulas (Equations (2)–(4)) was systematically corrected to (h′) through an iterative calibration process (Figure 7), ensuring alignment with the measured temperature data.

#### 3.2.3. Model Prediction Results

**Figure 8:**
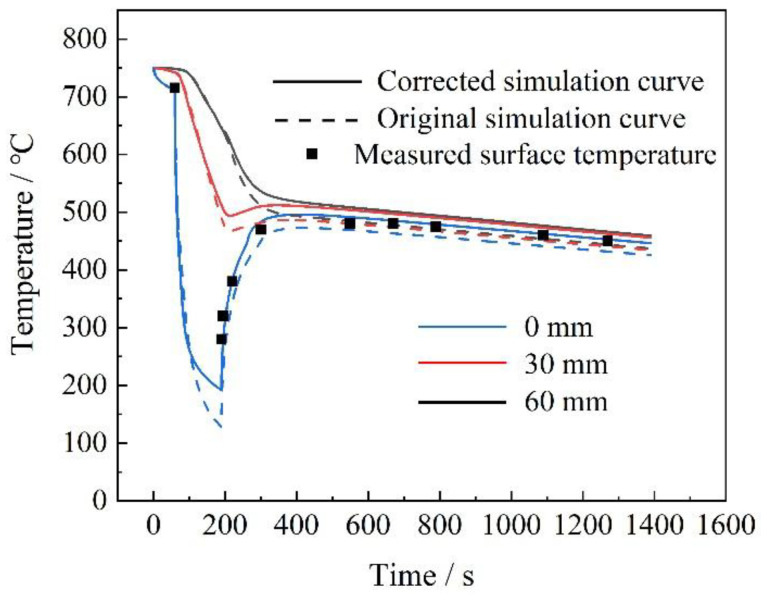
Predicted temperature curve.

The model calculated the temperature curves at the surface (0 mm depth) and at depths of 30 and 60 mm within the thick plate during the cooling stage, as illustrated in Figure 8. In the figure, the dashed lines represent the temperature curves predicted by conventional methodologies, the solid lines represent the temperature curves after the adjustment of the convective heat transfer coefficient, and the black points represent the on-site measured temperature points. After adjusting the heat transfer coefficient, the model accurately predicted the surface temperature changes, closely matching the on-site process-tracking data. After correcting h with the return temperature data, the simulation’s R² increased from 0.72 to 0.96, indicating that our method is more accurate.

According to this model, the cooling rate at a depth of 10 mm from the surface of the thick plate was 8.0 °C/s, while at the 1/4 thickness position, it was 2.1 °C/s, and at the core position, it was 1.1 °C/s. These refined predictions provided a more accurate representation of the cooling behavior of the thick plate during the TMCP process, which was essential for optimizing the process parameters and ensuring the desired mechanical properties of the final product.

**Figure 9:**
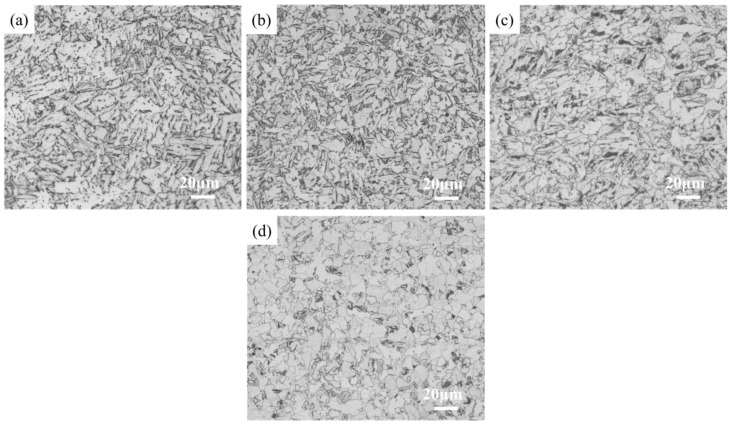
Microstructures under different cooling conditions: (**a**) 10 mm, (**b**) 8° C/s, (**c**) 60 mm, (**d**) 1.1 °C/s.

Continuous cooling tests were conducted on the experimental steel using cooling rates identical to those at 10 mm and 60 mm depths from the surface of the thick plate (8.0 °C/s and 1.1 °C/s, respectively), resulting in the microstructures shown in Figure 9b,d. Both the 10 mm depth position in the thick plate and the 8.0 °C/s continuous cooling condition predominantly exhibited bainitic structures. The microstructures at 60 mm depth and under 1.1 °C/s cooling consisted of ferrite, pearlite, and bainite. Laboratory-simulated specimens under model-calculated cooling rates showed high consistency in microstructure characteristics with actual thick plate specimens, demonstrating that the cooling rates derived from the model possess valuable reference significance for predicting microstructural evolution in thick plates.

### 3.3. Cooling Optimization

#### 3.3.1. Optimization of Process Simulation

According to the thick plate cooling model, the parameters that affected the prediction results included the convective heat transfer coefficient of the thick plate surface, initial temperature, and cooling time. Given the constraints of actual production equipment, where further increasing the water-cooling intensity is impractical, and considering that excessively rapid surface cooling could lead to a coarse microstructure and reduced toughness, in this study, we only investigated the effects of changing the cooling time and initial temperature on the cooling situation of a thick plate. The simulated process conditions are listed in Table 4, where S0 represents the original process with a surface temperature of 750 °C at the start of cooling and a cooling time of 130 s. The S1 process extended the cooling time to 160 s, while the S2 process extended the cooling time to 160 s and also increased the starting cooling temperature to 725 °C. The thick plate cooling model described in Section 3.2 was used to predict the cooling conditions.

**Figure 10:**
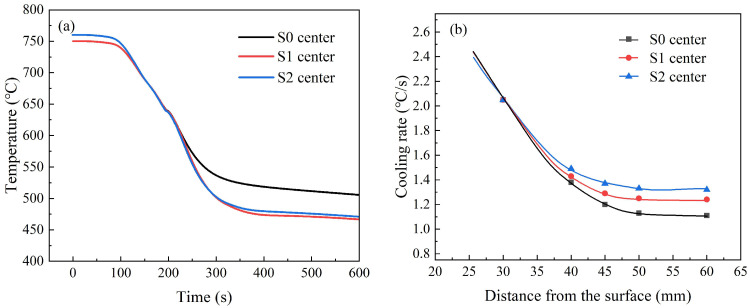
Simulation results: (**a**) temperature and (**b**) cooling rate.

Figure 10 presents the simulation results for the three different processes. Figure 10a presents the simulated cooling curves at a depth of 60 mm under three different processes. The reheat temperature was lower than that of S0, and the slope of the S2 curve was slightly higher than those of S0 and S1. When the temperature at a depth of 60 mm in the thick plate reached 720 °C, the temperature at a depth of 30 mm for processes S0 and S1 was 650 °C, while for the S2 process, it decreased to 615 °C. This observation indicates that the S2 process induced a steeper thermal gradient within the plate.

The average cooling rates at different depths are summarized in Figure 10b. Within the 30–60 mm depth range, the S2 process exhibited the highest cooling rate (1.36 °C/s), surpassing those of S1 (1.24 °C/s) and the baseline S0 (1.10 °C/s). Notably, both the S1 and S2 processes improved the 60 mm depth cooling rate compared with S0, with S2 achieving a 21.4% enhancement (from 1.10 °C/s to 1.36 °C/s).

**Figure 11:**
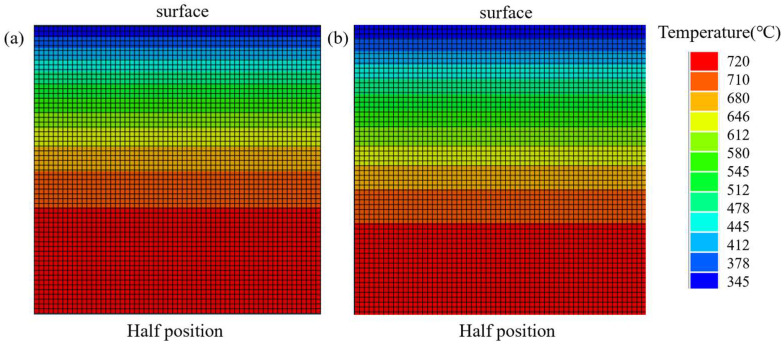
Temperature distribution across the thick plate cross-section at the onset of core phase transformation (720 °C): (**a**) S0 process, (**b**) S2 process.

Figure 11 shows the temperature distribution across the thick plate cross-section when the core region initiates phase transformation (720 °C) under the S0 and S2 processes. The blue and green regions in Figure 11b are significantly larger than those in Figure 11a, indicating that the S2 process exhibits a higher temperature gradient in the thick plate cross-section than the S0 process. This enhanced thermal gradient contributes to improving the cooling rate in the core region.

#### 3.3.2. Process Optimization and Pilot Production

**Figure 12:**
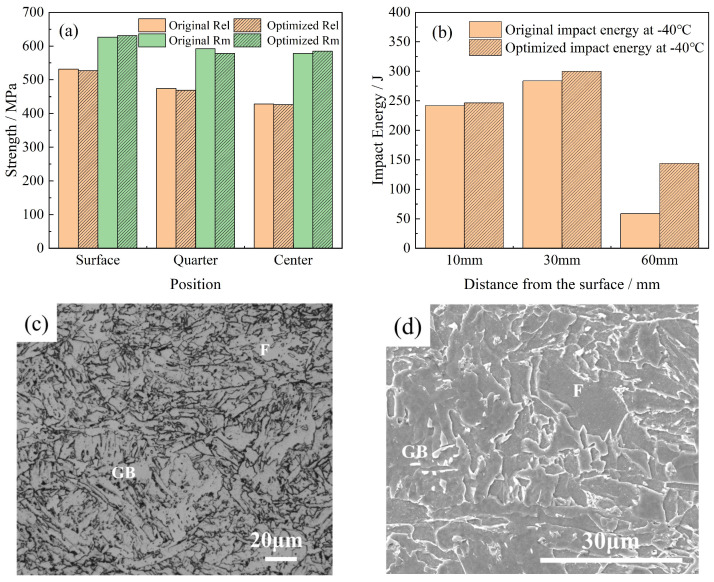
Mechanical properties and microstructures at optimized cooling parameters (S2): (**a**) Mechanical properties, (**b**) impact energy, (**c**,**d**) OM and SEM microstructures.

The S2 process was employed for the industrial trial production of S460 thick plates in this study. Samples were obtained from the same positions of the thick plates produced using the S2 process and the original process for mechanical property testing and microstructure observation. The comparative results are presented in Figure 12. No significant change in strength was observed before and after the optimization. In addition, the impact toughness at the surface and 1/4 positions showed no noticeable difference; however, the impact toughness at the core position was significantly improved, with a Charpy impact energy value of 144 J at −40 °C. Figure 12c,d presents the microstructure at the core position of the optimized process, indicating that the predominant microstructures were ferrite (F) and granular bainite (GB). Compared to Figure 5e,f, the optimized process produced almost no pearlite.

**Figure 13:**
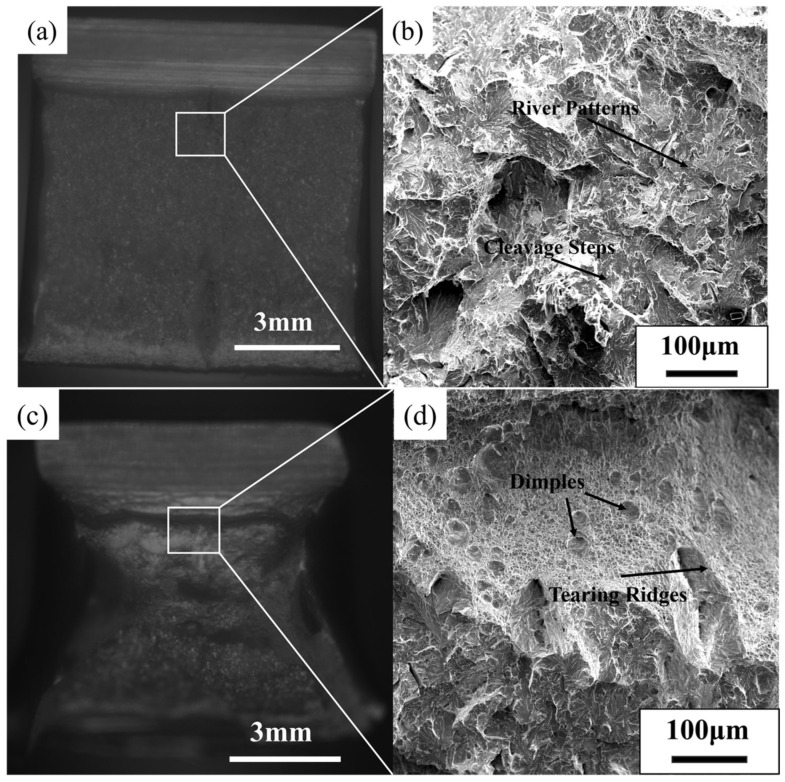
Impact fracture morphology at the center of the S460 thick plate: (**a**,**b**) Original process; (**c**,**d**) Optimized process.

Figure 13 presents the low-temperature impact fracture morphology at a 60 mm depth in the thick plate, observed via scanning electron microscopy (SEM). As shown in Figure 13a,b, the fracture surfaces produced by the original process are smooth and highly reflective. The crack initiation zone exhibits abundant river patterns and cleavage steps under SEM, which are characteristic of a typical ductile fracture.

In contrast, Figure 13c,d reveals significant deformation of the impact fracture surfaces after process optimization. SEM observations reveal ductile fracture features, including dimples and tear ridges, alongside a small number of residual river patterns. These findings indicate that the fracture mechanism transitions from brittle to ductile during the optimized process.

## 4. Discussion

### 4.1. Analysis of Factors Contributing to Increased Core Cooling Velocity

According to the law of conservation of energy, the temperature change at a 60 mm depth in the thick plate was the result of both heat transfer and internal heat generation. During the cooling process, internal heat generation was the latent heat of phase transformation, the amount of which was determined by the material’s inherent properties. Heat transfer, however, was influenced by the internal temperature gradient of the thick plate, where the greater the temperature gradient, the more heat was transferred per unit time, resulting in a faster cooling rate.

**Figure 14:**
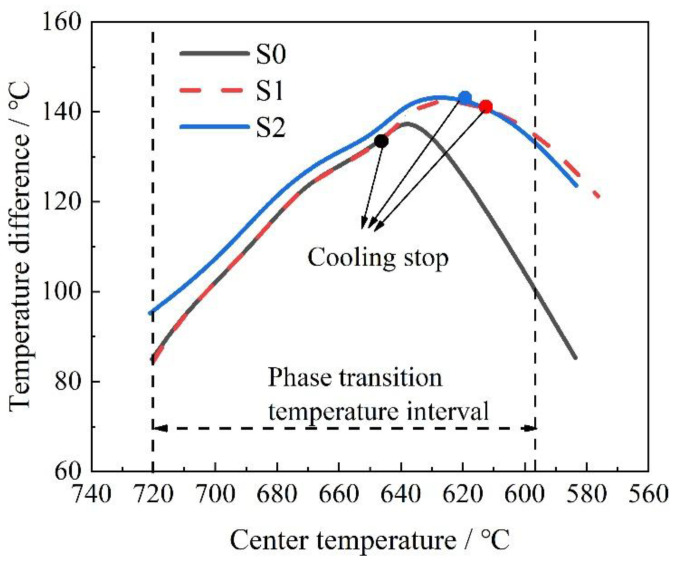
Temperature difference diagram between the core of thick plate and 1/4 position.

Based on the modeling results, the relationship curve between the temperature difference between 30 mm and 60 mm depth from the surface (ΔT_30–60_) and the core temperature at 60 mm depth (T_60_) was plotted (Figure 14) to represent the internal temperature gradient variation during thick plate cooling. The data points in the figure indicate the cooling-termination moment. The results demonstrate that the ΔT_30–60_ values of Process S2 before cooling termination exceed those of Processes S0 and S1. When the temperature decreases below 640 °C, both Processes S1 and S2 exhibit higher ΔT_30–60_ values than Process S0.

The findings reveal that increasing the initial cooling temperature effectively enhances the internal cooling gradient of thick plates. Moreover, at equivalent core temperatures (T_60_), other positions in the S2-processed plate maintain lower temperatures than those in the S0 and S1 processes, resulting in greater latent heat release during the phase transformation. This enhanced latent heat release further mitigates the adverse effects of phase transformation heat on core cooling. Additionally, extended cooling prevents premature gradient reduction, maintaining core cooling rates during phase transformation. Consequently, the combined strategy of elevating the initial cooling temperature and extending the cooling duration can significantly improve the cooling rate in the core region of thick plates.

Therefore, increasing the initial cooling temperature and extending the cooling time enhanced the cooling rate at the 60 mm depth of the thick plate. The higher starting cooling temperature allowed the 60 mm depth to undergo a longer period of water cooling before entering the phase transformation temperature range, which helped to increase the internal temperature gradient of the thick plate. This also enabled more latent heat to be released from other parts of the plate before the 60 mm depth underwent a phase transformation, thereby reducing the impact of the phase transformation latent heat on the 60 mm depth cooling. Extending the cooling time ensured that the 60 mm depth of the thick plate maintained a higher cooling rate within the phase transformation temperature range, thereby improving the microstructure at a depth of 60 mm.

### 4.2. Microstructure Evolution Mechanism

The cooling rate at different positions within a thick plate significantly influences its microstructure. In the original process, at a depth of 60 mm, the slower cooling rate and higher transformation temperature allowed the carbon atoms to diffuse. As the temperature of the overcooled austenite decreased, some ferrite began to precipitate. A faster cooling rate resulted in a greater degree of overcooling, thereby reducing the initial ferrite precipitation. Carbon atoms diffused toward the ferrite boundaries, increasing the carbon content in the overcooled austenite.

As the cooling rate increased, the transformation temperature decreased, thereby limiting the diffusion of carbon atoms. At the ferrite boundaries, the carbon atom concentration was insufficient to precipitate carbides, which led to the formation of granular bainite. As shown in Figure 5c,d and Figure 12c,d, granular bainite consisted of a ferrite matrix and island-like M-A constituents. The morphology and size of these M-A islands were affected by the transformation temperature. At higher transformation temperatures, the M-A constituents were larger, while at lower temperatures, smaller constituents were formed.

### 4.3. Toughness Optimization Mechanism

Figure 12 shows the significant enhancement in the low-temperature toughness of the 60 mm depth region of the thick plate produced using the optimized process, with negligible changes in strength. Consequently, in this study, we investigated the mechanisms underlying the improved low-temperature toughness at a 60 mm depth in a thick plate after optimization, focusing on factors such as the microstructure content, carbide distribution, high-angle grain boundary content, and grain orientation.

#### 4.3.1. Influence of Microstructure Content on Toughness

**Figure 15:**
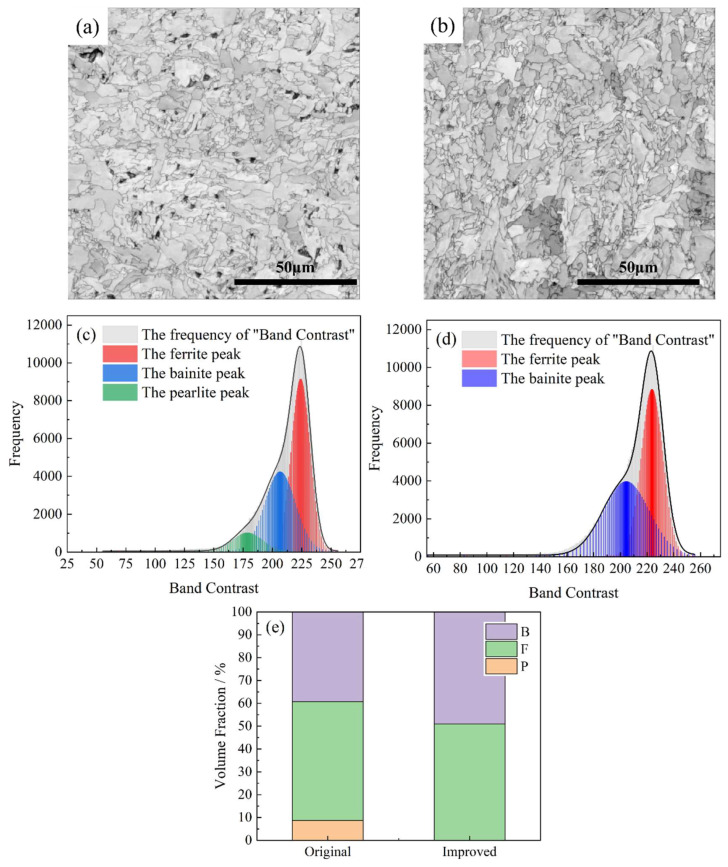
Distribution of microstructure types in the 60 mm depth of the thick plates: (**a**) original BC map, (**b**) optimized BC map, (**c**) original BC peak fitting, (**d**) optimized BC peak fitting, (**e**) Distribution of microstructure types.

Figure 15 presents the Kikuchi band contrast (BC) maps of the 60 mm depth region of the thick plate under EBSD analysis before and after the process optimization. In these maps, areas with more lattice distortions and dislocations, which are indicative of defects, exhibit lower Kikuchi band intensities and appear darker. Conversely, the areas with fewer defects appeared lighter. Ref. [29] Ferrite, which had the lowest carbon content, appeared lightest in the maps due to its higher transformation temperature and lower dislocation density. In contrast, pearlite, with its high carbon atom content and significant lattice distortion, appeared the darkest, while bainite fell between the two in terms of appearance. The 60 mm depth microstructure composition was calculated using peak fitting, as shown in Figure 15c. In the original process, the proportions of bainite, ferrite, and pearlite were 39.3, 52.0, and 8.7%, respectively. After process optimization, pearlite was nearly eliminated, while bainite and ferrite accounted for 49.1 and 50.9%, respectively. The pearlite microstructure consisted of fine cementite particles and a ferrite matrix. Under stress, the spaces between the cementite particles were prone to cracking, leading to crack initiation or propagation, which was detrimental to the low-temperature toughness. Therefore, the avoidance of pearlite transformation at a depth of 60 mm due to the increased cooling rate was one of the reasons for the enhanced low-temperature toughness in the optimized process.

#### 4.3.2. Influence of M-A Islands on Low-Temperature Toughness

**Figure 16:**
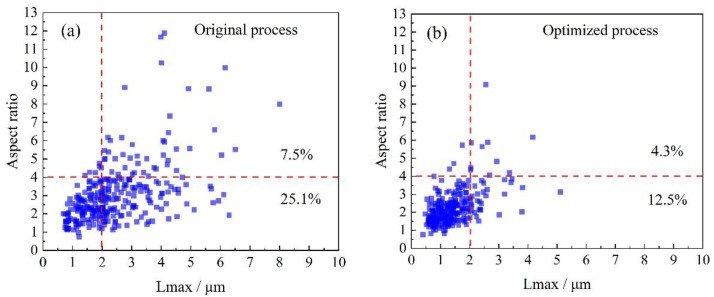
Statistical analysis of the shape and size of M-A island in the 60 mm depth of the thick plates (**a**) original process, (**b**) optimized process.

The optimized thick plate contains a substantial amount of granular bainite in its central region (Figure 12). The microstructure is characterized by martensite/austenite (M-A) islands dispersed within the ferrite matrix. These M-A constituents, formed through phase transformation from carbon-enriched retained austenite, typically consist of twinned martensite with a portion of the retained austenite phase. The morphology and size of the M-A islands in granular bainite significantly affected the low-temperature toughness of the material [30]. The dimensions and morphologies of the M-A islands, as statistically analyzed from the SEM images, are presented in Figure 16. After process optimization, the proportion of large blocky M-A islands (Lmax > 2 μm, Lmax/Lmin < 4) decreased from 25.1 to 12.5%, while the proportion of elongated M-A islands (Lmax > 2 μm, Lmax/Lmin > 4) decreased from 7.5 to 4.3%. Smaller M-A constituents delay crack initiation by homogenizing stress distribution [30]. Therefore, the impact of fine M-A islands on low-temperature toughness is almost negligible. However, coarse M-A islands are prone to self-fracture or separation from the matrix under stress, leading to the formation of microcracks and reduced toughness. Therefore, the decreased proportion of large M-A islands at a 60 mm depth in the thick plate produced through the optimized process improved its low-temperature toughness.

#### 4.3.3. Influence of Misorientation Angles on Toughness of Thick Plate S460

**Figure 17:**
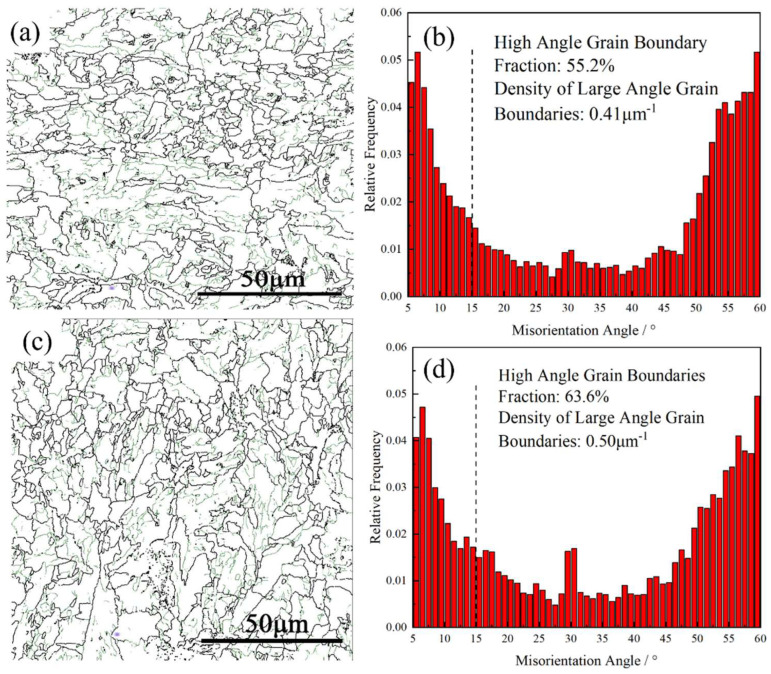
Grain boundary of the 60 mm depth: (**a**) original Grain boundary map, (**b**) original Grain boundary frequency distribution, (**c**) optimized Grain boundary map, (**d**) optimized Grain boundary frequency distribution.

Figure 17 presents the grain boundary distribution maps of the 60 mm depth region in the thick plate before and after process optimization, along with the relative frequency statistics of the different angle grain boundaries. Grain boundaries with an angle greater than 15° were generally considered high-angle grain boundaries and are represented by black solid lines in the figure. Grain boundaries with an angle of less than 15° were considered low-angle grain boundaries and are represented by green solid lines in the figure. According to the statistics, after process optimization, the proportion of high-angle grain boundaries at the 60 mm depth position increased from 55.2 to 63.6%, while the density of high-angle grain boundaries increased from 0.41 to 0.5 μ^−1^.

The fracture modes of materials under impact loads are categorized as transgranular and intergranular fractures. The increase in the content of high-angle grain boundaries led to grain refinement, which reduced the dislocation pile-up within the grains and decreased the stress concentrations, making transgranular fracture less likely to occur. Moreover, the increased density of high-angle grain boundaries results in a greater deflection angle of cracks during intergranular fracture, which is detrimental to crack propagation [9]. Therefore, the higher density of high-angle grain boundaries contributed to the improved low-temperature toughness.

#### 4.3.4. Influence of Grain Orientation on Toughness

**Figure 18:**
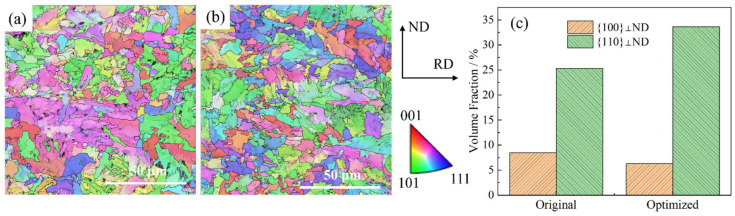
Statistics of grain orientation: (**a**)original IPF map, (**b**) optimized IPF map, (**c**) contrast of grain orientation.

Figure 18 compares the crystallographic orientation maps and corresponding statistical distributions of the {100} and {110} planes in the thick plate’s 60 mm depth region along the rolling direction before (S0) and after (S2) process optimization. Quantitative analysis revealed a marginal reduction in the {100} planar fraction coupled with a 25% enhancement in the {110} planar dominance following optimization. This microstructural evolution is particularly significant for body-centered cubic (BCC) metals, where {100} planes serve as preferential cleavage planes for brittle fractures [11].

To systematically evaluate the effect of grain orientation on crack resistance, we conducted a Schmid factor statistical analysis (n = 500 grains) for the predominant {110}<111> slip systems. The optimized process exhibited a superior average Schmid factor of 0.43 (S2) compared to 0.37 (S0) in the baseline condition, representing a 15% improvement in the slip system activation capability under external loading. This enhancement correlates with the improved deformation behavior of the {110}-oriented grains, which demonstrate three key advantages: (1) enhanced dislocation slip propensity, (2) reduced dislocation entanglement likelihood, and (3) diminished stress concentration susceptibility. These combined effects effectively inhibit both crack initiation and propagation mechanisms.

Consequently, the process-optimized microstructure characterized by enhanced {110} planar dominance demonstrates superior impact toughness through improved slip compatibility and reduced stress concentrations.

## 5. Conclusions

This study examined the microstructural and property variations in 120 mm-thick S460 plates fabricated by TMCP. Based on data from on-site process tracking, a heat transfer model was developed to predict the temperature curve during the TMCP cooling phase. The TMCP cooling procedure was subsequently used in this model. The following conclusions were drawn:

(1) The original TMCP-processed thick plate exhibited a bainite-ferrite-dominant microstructure with 8.7% pearlite formation at the 60 mm depth (60 mm depth). This caused severe toughness degradation, showing a −40 °C impact energy of 59 J at the 60 mm depth versus 242 J at 30 mm (quarter depth).
(2) The calibration of the convective heat transfer coefficients using surface temperature recovery data improved the model accuracy. Simulations revealed a 60 mm depth cooling rate of 1.10 °C/s for the conventional processing. Optimized parameters (initial cooling temperature: 715 → 725 °C; cooling duration: 130 → 160 s) elevated the 60 mm depth cooling rate to 1.36 °C/s.
(3) The optimized process enhanced the 60 mm depth toughness to 144 J (−40 °C), attributed to:
  - Pearlite suppression (8.7% to <1%)
  - M-A constituent refinement (50% reduction in large blocky M-A)
  - Increased high-angle grain boundary density (0.41 to 0.5 μm^−1^)
  - Elevated {110} texture component.

## Figures and Tables

**Table 1:** Chemical composition of S460 (weight percentage, wt%).

C	Si	Mn	P	S	Al	Cr	Nb	Ti	Ni	Cu	B
0.07	0.22	1.57	0.009	0.0014	0.03	0.17	0.041	0.012	0.63	0.21	0.0003

**Table 2:** Thermophysical properties of the material [28].

t (°C)	100	200	300	400	500	600	700	800	900
λ (W·m^−1^·k^−1^)	44.3	45.1	46.2	44.8	40.2	36.1	36.5	37.5	38.4
c (KJ·kg^−1^·K^−1^)	525	575	610	627	640	652	662	669	675
*h* (N/s/mm/°C)	5.9	4.4	3.3	2.4	2.1	1.5	9.9	8.5	6.3
*h′* (N/s/mm/°C)	2.3	2.8	3.3	2.5	2.2	1.9	1.6	1.5	1.2

Note: *h*: Convective heat transfer coefficient calculated by empirical formulas (Equations (2)–(4)) during water cooling. *h′*: Corrected convective heat transfer coefficient after iterative adjustment based on temperature recovery data (Section 3.2.2).

**Table 3:** Recorded data of return-back temperature.

Time (s)	0	5	30	120	360	480	600	900	1080
Temperature (°C)	282	320	375	461	473	471	470	460	458

**Table 4:** Simulation process.

Number	Cooling Temperature (°C)	Cooling Time (s)
S0	715	130
S1	715	160
S2	725	160

## Data Availability

The original contributions presented in this study are included in the article. Further inquiries can be directed to the corresponding author.
